# Coagulation Disorders in Patients With Acute Respiratory Distress Syndrome Following Acute Aortic Dissection: A Prospective Observational Study

**DOI:** 10.31083/RCM36372

**Published:** 2025-08-25

**Authors:** Maomao Liu, Tengke Wang, Yan Yu, Xuran Lu, Zheyuan Chen, Li Yu, Sihao Cheng, Lingru Li, Liangshan Wang, Nan Liu

**Affiliations:** ^1^Center for Cardiac Intensive Care, Beijing Anzhen Hospital, Capital Medical University, 100029 Beijing, China

**Keywords:** acute aortic dissection, acute respiratory distress syndrome, coagulation, fibrinolysis, cardiopulmonary bypass

## Abstract

**Background:**

Coagulation disorders are potentially one of the most important pathogeneses of acute respiratory distress syndrome (ARDS) following acute type A aortic dissection (ATAAD). This study aimed to determine whether aortic dissection singularly and cardiopulmonary bypass (CPB) surgery can activate coagulation pathways, promoting ARDS development in patients with ATAAD.

**Methods:**

A total of 450 patients who received treatment at Beijing Anzhen Hospital, Capital Medical University, between March 2023 and February 2024 were consecutively enrolled in this prospective cohort study. We analyzed the clinical factors and measured serum coagulation biomarkers by enzyme-linked immunosorbent assay (ELISA) among patients with ATAAD, aortic aneurysm (AA), or unstable angina (UA). Logistic regression, two-way analysis of variance (ANOVA), and Spearman's correlation analysis were performed. Furthermore, the patients with ATAAD were divided into ARDS (based on chest radiographic findings and an oxygenation index ≤300 mmHg) and non-ARDS groups for subgroup comparisons.

**Results:**

The incidence of postoperative ARDS among patients with ATAAD was 20.7% (13.3% in the AA group and 7.3% in the UA group). Preoperatively, prothrombin time (PT) was longer in patients with ATAAD than in those with AA or UA ((odds ratio (OR): 12.0, 95% confidence interval (CI): 11.5–12.6) vs. (OR: 11.4, 95% CI: 10.9–12.1) vs. (OR: 11.2, 95% CI: 10.8–11.6), respectively; *p* < 0.001). The D-dimer levels, fibrin degradation products (FDPs), factor XIIa, and factor VIII-Ag (FVIII-Ag) were significantly elevated preoperatively and postoperatively in patients with ATAAD. The FDP levels in the ATAAD subgroup immediately after surgery were significantly higher in the ARDS group compared with those in the non-ARDS group (OR: 2.26, 95% CI: 1.13–4.54; *p* = 0.022). In addition, a negative correlation existed between the FXII level (correlation coefficient r = –0.682, *p* = 0.043) at 24 hours after surgery and the oxygenation index.

**Conclusion:**

Coagulation activation may be caused by aortic dissection singularly and CPB, which promotes postoperative ARDS in patients with ATAAD.

## 1. Introduction

Acute lung injury (ALI) and acute respiratory distress syndrome (ARDS) are common complications that may occur during perioperative management, especially in high-risk populations of patients undergoing surgery [1, 2, 3]. ARDS is a primary cause of postoperative acute hypoxemic respiratory failure and its etiologies are diverse [4, 5, 6, 7]. The occurrence of ARDS has also been investigated following cardiac surgery (CS) involving cardiopulmonary bypass (CPB) procedures [3, 7, 8, 9]. Despite the continual refinement of perioperative management and surgical techniques, up to 20% of patients are affected by this postsurgical complication [3, 10]. Postoperative ARDS can seriously affect patient outcomes, with mortality rates as high as 40–80% in severe cases [2, 7, 11, 12] and functional limitations sometimes persisting 5 years after an ARDS episode [13].

Although the pathogenesis of ARDS is not entirely clear, recent studies suggest that the activation of coagulation is closely related to the development of ARDS [6, 14, 15, 16, 17, 18] and blockade of the initiating steps of the clotting cascade may be beneficial for patients with ARDS [6, 19]. During cardiac surgery, CPB procedures and extensive surgical trauma can induce the widespread activation of the coagulation system [17, 20, 21], and the pathogenesis of acute type A aortic dissection (ATAAD) has been shown to be associated with coagulation disorders [22]. Therefore, both ATAAD itself and CPB can contribute to the development of multiple disorders of the coagulation system; however, there is little data supporting the activation of coagulation cascade plays a role in the etiology of ARDS after aortic surgery.

The occurrence of ALI/ARDS following aortic surgery is unpredictable, although recent studies have suggested that early identification and improved perioperative care can prevent postoperative ALI/ARDS. Although several risk factors have been identified are associated with the occurrence of post-operative ARDS, to our knowledge, barely any study has illustrated the association between coagulation markers and ARDS in ATAAD patients. Therefore, the aim of this study was to explore the correlations between the serum levels of various biomarkers of coagulation and the occurrence of ARDS following aortic surgery, as these markers could serve as reliable predictors of the risk of ALI/ARDS.

## 2. Methods

### 2.1 Design and Participants 

This single-center prospective observational study was performed at the Center for Cardiac Intensive Care of Beijing Anzhen Hospital, Capital Medical University. A total of 450 patients were consecutively enrolled between March 2023 and February 2024, including 150 patients diagnosed with ATAAD, 150 with aortic aneurysm (AA), and 150 with unstable angina (UA). Patients with ATAAD and AA underwent Sun’s surgery or partial aortic arch replacement, whereas patients with UA underwent off-pump coronary artery bypass grafting (OPCABG).

All adult subjects were over 18 years of age, had been diagnosed with AA (chronic onset), UA and ATAAD (with a time from onset of under two weeks and had been scheduled for emergency surgery). The exclusion criteria were as follows: patients with neoplastic or chronic coagulation and inflammatory disorders; patients with missing specimens or clinical data; and patients who experienced a failed surgical intervention or died within 24 hours after surgery. Clinical data of these patients were collected during the hospitalization period. In addition, nine patients in each group were randomly selected and venous plasma was collected and subjected to enzyme-linked immunosorbent assays (ELISAs) to quantify various markers of coagulation and inflammation. We randomly selected 9 patients from each group by using a random number generator to ensure the reproducibility and validity of the findings.

### 2.2 Definitions

All subjects underwent contrast-enhanced computed tomography (CT) to confirm the surgeons’ diagnoses of ATAAD [23]. Based on the European Society of Intensive Care Medicine (ESICM) definition [5], ARDS was confirmed via arterial blood gas analysis and chest radiography. These criteria included an acute onset, an oxygenation index (partial pressure of oxygen in arterial blood/fraction of inspired oxygen, PaO_2_/FiO_2_) ≤300 mmHg for ARDS, regardless of the ventilator settings, the appearance of bilateral pulmonary infiltration on chest radiography, and the presence of respiratory failure that could not be fully explained by cardiac failure or fluid overload. Hypertension (HTN) is defined as having a systolic blood pressure (SBP) of ≥140 mmHg and/or a diastolic blood pressure (DBP) of ≥90 mmHg when no antihypertensive drugs are used. The diagnosis of diabetes is based on the recommended standards of the American Diabetes Association (ADA) in 2010. Acute kidney injury (AKI) was confirmed by the guideline published by the Kidney Disease Improving Global Outcomes (KIDGO) in 2012. Heart failure (HF) was defined as left ventricular ejection fraction ≤40% accompanied by symptoms and/or signs. Chronic lung diseases (CLD) include chronic obstructive pulmonary disease, bronchiectasis, asthma or tuberculosis, etc. Neurological disease (ND) refer to ischemic/hemorrhagic stroke, brain tumors or brain trauma. Altered consciousness was defined as drowsiness, stupor or coma that occurs before surgery due to various reasons. Chest radiography was performed daily, and the results were interpreted by two radiologists. Blood gas levels were measured every 4 hours. The primary outcome variable was the development of ARDS within two days after surgery.

### 2.3 Anesthesia and Surgery

Radial artery pressure, dorsalis pedis artery pressure, and central venous pressure were monitored before surgery in all patients using established methods. Anesthesia was administered in accordance with institutional standards. Aortic surgical procedures were performed under general anesthesia, CPB, deep hypothermic circulatory arrest (DHCA), and selective cerebral perfusion were conducted using a modified elephant trunk technique (Sun’s procedure) as previously described [22].

### 2.4 Clinical Data and Blood Samples

Data related to clinical characteristics, demographic information, medical history, intraoperative variables, and details pertaining to the subsequent course of recovery in the intensive care unit (ICU) post-surgery were recorded. Each patient’s preoperative risk profile was evaluated using the European System for Cardiac Operative Risk Evaluation (EuroSCORE) [24]. The dosage of vasoactive drug administration and organ function were assessed during the first 48 h post-surgery using the vasoactive inotrope score (VIS) [25] and the Sequential Organ Failure Assessment (SOFA) scale [26], respectively.

Blood samples were collected in sodium citrate tubes through a central venous catheter at the following three time points: immediately prior to anesthesia induction; immediately after surgery; and 24 hours after surgery. Samples were centrifuged at 1550 × g for 15 min at 4 °C, and the supernatants were aliquoted and stored at –80 °C for later analysis. Serum levels of factor XII (FXII), factor XIIa (FXIIa), factor VIII-related antigen (FVIII-Ag), plasmin-antiplasmin complex (PAP), interleukin one beta (IL-1β), and tumor necrosis factor-alpha (TNFα) were assayed using ELISA kits (USCN KIT INC, Wuhan, China).

### 2.5 Statistical Analysis

We used the median to interpolate the missing data of continuous variables and conducted the Kolmogorov-Smirnov test. Continuous variables were expressed as the mean ± standard deviation and were analyzed using a Student’s *t*-test for normally distributed data; alternatively, variables with a skewed distribution were expressed as medians and quartiles and were analyzed using a Mann-Whitney U-test. Categorical variables were expressed as numbers and percentages, and a χ^2^ test or Fisher’s exact test was used to compare groups as appropriate.

The baseline characteristics of the ATAAD group were first compared with those of the AA group and UA group separately, followed by ANOVA across all three groups with Bonferroni correction for multiple comparisons. Patients with ATAAD were further stratified into ARDS and Non-ARDS subgroups, with a comparative analysis conducted on their baseline characteristics. All coagulation biomarkers were normalized and standardized using log- and z-score transformations. Logistic regression models were applied separately in the ATAAD group, AA group and UA group to assess the associations between coagulation biomarkers and postoperative ALI/ARDS, with odds ratios (ORs) and 95% confidence intervals (95% CIs) calculated. Model 1 was a univariate model and Model 2 was adjusted for traditional confounders, including sex, age, body mass index (BMI), HTN, diabetes, AKI, HF, liver dysfunction (LD), CLD, ND, smoking status, alcohol consumption, prior CS, preoperative aspartate aminotransferase (AST) level, and the preoperative oxygenation index (PaO_2_/FiO_2_, P/F). These covariates include medical history data and some preoperative meaningful indicators (AST and P/F) between the ARDS group and Non-ARDS group in the baseline characteristics (**Supplementary Tables 1,2**). Collinearity test was conducted in the multivariate analysis (**Supplementary Table 3**). A two-way ANOVA was used to account for both group and time as independent variables in ELISA. Spearman correlation analysis was used to analyze the associations between variables for inferring coagulation activity and the oxygenation index.

All analyses were performed using R software 4.3.1 (R Foundation, Vienna, Austria) (https://www.r-project.org/) and Prism 10.2 (GraphPad Corp, San Diego, CA, USA). *p*-value < 0.05 indicates statistical significance (*p* < 0.017 indicates statistical significance after Bonferroni correction).

## 3. Results

### 3.1 Subject Preoperative Data

Baseline and preoperative laboratory values of the participants are presented in Table 1. Patients in the ATAAD group were younger than those in the AA group (49.1 ± 11.5 years), whereas the patients with ATAAD exhibited a higher incidence of elevated blood pressure (84% vs 62.7%, respectively). Preoperatively, patients with ATAAD presented with significantly higher D-dimer levels than did those in either the AA group (2589 [978.5–7919] vs 180 [68.3–670.8] ng/mL; *p* < 0.001) or the UA group (2589 [978.5–7919] vs 92.5 [56.0–157.0] ng/mL; *p* < 0.001). The levels of FDPs in the ATAAD group increased significantly at admission (27.5 [10.9–69.3] vs 1.4 [0.5–4.6] µg/mL, *p* < 0.001; 27.5 [10.9–69.3] vs 0.8 [0.5–1.2] µg/mL, *p* < 0.001). However, the levels of fibrinogen (FBG) in the ATAAD group decreased significantly before surgery (2.3 [1.8–2.9] vs 2.9 [2.4–3.4] g/L, *p* < 0.001; 2.3 [1.8–2.9] vs 3.1 [2.6–3.6] g/L, *p* < 0.001).

**Table 1.** **Baseline among three groups**.

Variables		ATAAD	AA	UA	^1^ *p*	^2^ *p*	^3^ *p*
		(n = 150)	(n = 150)	(n = 150)			
Demographic information and preoperative complications							
	Age (yrs)	49.1 ± 11.5	55.3 ± 13.5	62.8 ± 8.1	< **0.001**	< **0.001**	< **0.001**
	Sex (male%)	114 (76.0)	109 (72.7)	83 (55.3)	0.597	< **0.001**	< **0.001**
	BMI (kg/m^2^)	27.2 ± 3.8	25.6 ± 3.5	25.6 ± 3.1	< **0.001**	< **0.001**	< **0.001**
	Smoking	65 (43.3)	72 (48.0)	49 (32.7)	0.487	0.074	**0.022**
	Alcohol	19 (12.7)	42 (28.0)	33 (22.0)	**0.002**	**0.047**	**0.004**
	HTN	126 (84.0)	94 (62.7)	90 (60.0)	< **0.001**	< **0.001**	< **0.001**
	Diabetes	13 (8.7)	9 (6.0)	57 (38.0)	0.506	< **0.001**	< **0.001**
	AKI	23 (15.3)	7 (4.7)	5 (3.3)	**0.004**	**0.001**	< **0.001**
	LD	11 (7.3)	7 (4.7)	4 (2.7)	0.466	0.112	0.171
	HF	49 (32.7)	57 (38.0)	88 (58.7)	0.398	< **0.001**	< **0.001**
	CLD	4 (2.7)	2 (1.3)	4 (2.7)	0.680	1	0.664
	ND	1 (0.7)	4 (2.7)	4 (2.7)	0.367	0.367	0.360
	Prior CS	2 (1.3)	13 (8.7)	1 (0.7)	**0.008**	1	< **0.001**
	Shock	7 (4.7)	0 (0.0)	0 (0.0)	0.022	0.022	**0.001**
	AC	15 (10.0)	1 (0.7)	0 (0.0)	**0.001**	< **0.001**	< **0.001**
	EuroSCORE	5.0 (5.0, 6.0)	4.0 (3.3, 6.0)	2.0 (1.0, 3.0)	< **0.001**	< **0.001**	< **0.001**
Preoperative laboratory values							
	PaCO_2_ (mmHg)	36.7 (33.3, 39.7)	34.6 (32.3, 36.8)	34.6 (31.7, 36.8)	< **0.001**	< **0.001**	< **0.001**
	PaO_2_ (mmHg)	98.1 (76.4, 122.8)	89.7 (81.9, 95.0)	90.9 (80.9, 101.1)	**0.015**	0.092	**0.034**
	Lac (mmol/L)	1.5 (1.0, 2.3)	1.5 (1.1, 1.8)	1.7 (1.4, 2.1)	0.338	0.113	**0.004**
	PT	12.0 (11.5, 12.6)	11.4 (10.9, 12.1)	11.2 (10.8, 11.6)	< **0.001**	< **0.001**	< **0.001**
	APTT	30.3 (28.5, 32.3)	31.4 (29.6, 33.8)	31.0 (29.0, 33.2)	**0.001**	0.069	**0.004**
	D-dimer (ng/mL)	2589.0 (978.5, 7919.0)	180.0 (68.3, 670.8)	92.5 (56.0, 157.0)	< **0.001**	< **0.001**	< **0.001**
	FBG (g/L)	2.3 (1.8, 2.9)	2.9 (2.4, 3.4)	3.1 (2.6, 3.6)	< **0.001**	< **0.001**	< **0.001**
	FDPs (µg/mL)	27.5 (10.9, 69.3)	1.4 (0.5, 4.6)	0.8 (0.5, 1.2)	< **0.001**	< **0.001**	< **0.001**

^1^*p*, *p* value of ATAAD group vs AA group; ^2^*p*, *p* value of ATAAD group vs UA group; ^3^*p*, *p* value of one-way ANOVA among three groups; ATAAD, acute type A aortic dissection; AA, aortic aneurysm; UA, unstable angina; BMI, body mass index; PT, prothrombin time; APTT, activated partial thromboplastin time; HTN, hypertension; AKI, acute kidney injury; LD, liver dysfunction; HF, heart failure; CLD, chronic lung disease; CS, cardiac surgery; ND, neurological dysfunction; AC, altered consciousness; EuroSCORE, European System for Cardiac Operative Risk Evaluation; Lac, lactic acid; FBG, fibrinogen; FDPs, fibrinogen degradation products. ^1^*p* and ^2^*p* < 0.017 indicates statistical significance; ^3^*p* < 0.05 indicates statistical significance. The bolded data indicate statistical significance.

### 3.2 Comparison of Coagulation Biomarker Levels Between the ATAAD, AA, and UA Groups

The duration of the CPB procedure was significantly longer in the patients with ATAAD (179 [158–206] min) than it was in the other two groups (*p* < 0.001 for ATAAD vs. AA and for ATAAD vs. UA), as was the aortic cross-clamping time (96.5 [86–116] min; *p* < 0.001 for ATAAD vs. AA and for ATAAD vs. UA). Immediately after surgery, the PaO_2_ values (110.0 [85.5–160.5] vs. 151.5 [141.3–206.5] mmHg, ATAAD vs. AA group, *p* < 0.001; vs. 158.0 [120.5–205.8] mmHg, ATAAD vs UA group, *p* < 0.001) decreased and lactic acid (Lac) level increased more significantly in the ATAAD group than in the other two groups. The activated partial thromboplastin time (APTT) at the end of surgery was longer (33.9 [31.0–40.3] s) in the patients with ATAAD than that in the other groups (*p* = 0.001 for ATAAD vs. AA and for ATAAD vs UA). The D-dimer level in the ATAAD group increased significantly over the 24 hours post-surgery. Similarly, the trend in the levels of FDPs was consistent with that of the D-dimer levels (Table 2). More importantly, ARDS represented 13.8% of total patients and 20.7% of patients with ATAAD.

**Table 2.** **Intraoperative and postoperative clinical factors among three groups**.

Variables		ATAAD	AA	UA	^1^ *p*	^2^ *p*	^3^ *p*
		(n = 150)	(n = 150)	(n = 150)			
Surgery-related variables							
	Surgical duration (h)	8.0 (7.0, 9.0)	7.1 (6.0, 9.0)	4.5 (4.0, 5.0)	**0.001**	< **0.001**	< **0.001**
	CPB time (min)	179.0 (158.0, 206.0)	147.5 (123.0, 172.8)	0.0 (0.0, 0.0)	< **0.001**	< **0.001**	< **0.001**
	DHCA time (min)	20.0 (13.0, 30.0)	0.0 (0.0, 0.0)	0.0 (0.0, 0.0)	< **0.001**	< **0.001**	< **0.001**
	ACC time (min)	96.5 (86.0, 116.0)	86.5 (66.3, 100.8)	0.0 (0.0, 0.0)	< **0.001**	< **0.001**	< **0.001**
	Heparin input (mL)	10.0 (10.0, 10.0)	10.0 (7.6, 10.0)	0.0 (0.0, 0.0)	**0.005**	< **0.001**	< **0.001**
	Plasma input (mL)	0.0 (0.0, 400.0)	0.0 (0.0, 400.0)	0.0 (0.0, 0.0)	0.658	< **0.001**	< **0.001**
Postoperative laboratory values							
	HR	89.0 (75.0, 98.0)	84.0 (75.0, 89.8)	79.0 (70.0, 86.0)	0.032	< **0.001**	< **0.001**
	MAP (mm/Hg)	92.8 (83.0, 102.9)	80.0 (75.1, 86.7)	83.5 (74.3, 91.0)	< **0.001**	< **0.001**	< **0.001**
	ACT	160.0 (147.0, 176.0)	151.0 (141.3, 159.0)	153.0 (144.3, 163.0)	< **0.001**	**0.001**	< **0.001**
	PaCO_2_ (mm/Hg) 0 h	46.5 (42.6, 50.1)	43.5 (39.1, 46.1)	39.5 (36.2, 42.4)	< **0.001**	< **0.001**	< **0.001**
	PaCO_2_ (mm/Hg) 24 h	39.4 (35.4, 42.7)	39.8 (36.5, 42.9)	39.3 (35.6, 41.4)	0.565	0.214	0.169
	PaO_2_ (mm/Hg) 0 h	110.0 (85.5, 160.5)	151.5 (104.5, 206.5)	158.0 (120.5, 205.8)	< **0.001**	< **0.001**	< **0.001**
	PaO_2_ (mm/Hg) 24 h	93.2 (77.6, 115.8)	118.0 (95.1, 149.8)	127.0 (97.2, 153.0)	< **0.001**	< **0.001**	< **0.001**
	Lac (mmol/L) 0 h	2.4 (1.5, 3.5)	1.8 (1.3, 2.6)	1.2 (0.9, 1.5)	**0.001**	< **0.001**	< **0.001**
	Lac (mmol/L) 24 h	2.2 (1.6, 3.2)	3.2 (1.9, 5.0)	1.8 (1.2, 2.5)	< **0.001**	< **0.001**	< **0.001**
	P/F 0 h (%)	180.0 (134.1, 264.6)	275.0 (178.3, 375.3)	268.4 (203.7, 348.3)	< **0.001**	< **0.001**	< **0.001**
	P/F 24 h (%)	210.8 (151.4, 281.3)	289.0 (230.2, 371.3)	295.7 (242.0, 373.6)	< **0.001**	< **0.001**	< **0.001**
	NE (×10^9^/L) 24 h	10.0 (7.8, 12.4)	9.3 (7.0, 12.8)	9.7 (8.0, 12.0)	0.573	0.938	0.791
	NE (×10^9^/L) 48 h	13.6 (10.9, 16.5)	14.0 (10.2, 17.4)	11.8 (9.1, 14.9)	0.730	**0.001**	**0.001**
	PT 0 h	13.2 (12.4, 14.1)	13.0 (12.3, 14.2)	13.3 (12.5, 14.4)	0.628	0.344	0.354
	PT 24 h	13.0 (12.1, 13.9)	13.0 (12.3, 14.0)	13.5 (12.9, 14.5)	0.345	< **0.001**	< **0.001**
	APTT 0 h	33.9 (31.0, 40.3)	32.2 (29.2, 36.1)	31.5 (29.7, 35.4)	**0.001**	**0.001**	**0.001**
	APTT 24 h	30.4 (27.7, 33.9)	30.8 (28.6, 34.5)	31.0 (28.3, 34.1)	0.143	0.144	0.239
	D-dimer (ng/mL) 0 h	2532.0 (1180.8, 4083.5)	987.0 (420.5, 2798.8)	186.0 (112.3, 339.0)	< **0.001**	< **0.001**	< **0.001**
	D-dimer (ng/mL) 24 h	2294.5 (1173.0, 3390.8)	917.5 (469.0, 2651.5)	234.0 (156.3, 416.0)	< **0.001**	< **0.001**	< **0.001**
	FBG (g/L) 0 h	2.5 (2.0, 3.3)	2.3 (1.9, 2.9)	2.4 (1.8, 3.1)	0.043	0.091	0.091
	FBG (g/L) 24 h	3.7 (3.0, 4.5)	2.7 (2.3, 3.3)	3.1 (2.6, 3.7)	< **0.001**	< **0.001**	< **0.001**
	FDPs (µg/mL) 0 h	20.5 (10.3, 38.1)	7.9 (3.0, 21.2)	1.4 (0.9, 2.3)	< **0.001**	< **0.001**	< **0.001**
	FDPs (µg/mL) 24 h	22.1 (11.8, 35.3)	8.9 (4.6, 21.9)	1.9 (1.2, 3.3)	< **0.001**	< **0.001**	< **0.001**
	VIS 24 h	6.0 (3.0, 12.8)	5.0 (2.0, 11.0)	7.0 (4.0, 12.0)	0.050	0.909	0.070
	VIS 48 h	3.0 (0.0, 8.7)	0.0 (0.0, 7.0)	5.0 (0.0, 10.0)	**0.001**	0.815	**0.001**
	SOFA 24 h	10.0 (7.0, 13.0)	6.0 (5.0, 8.0)	5.0 (4.0, 7.0)	< **0.001**	< **0.001**	< **0.001**
	SOFA48 h	8.0 (5.0, 12.8)	5.0 (3.0, 6.0)	4.0 (3.0, 5.0)	< **0.001**	< **0.001**	< **0.001**
	ARDS (%)	31 (20.7)	20 (13.3)	11 (7.3)	0.124	**0.002**	**0.004**
	ICU duration (h)	68.5 (40.0, 151.8)	42.0 (19.0, 69.0)	25.5 (19.0, 48.0)	< **0.001**	< **0.001**	< **0.001**

^1^*p*, *p* value of ATAAD group vs AA group; ^2^*p*, *p* value of ATAAD group vs UA group; ^3^*p*, *p* value of one-way ANOVA among three groups; CPB, cardiopulmonary bypass; DHCA, deep hypothermic circulatory arrest; ACC, aortic cross-clamp; HR, heart rate at admission in intensive care unit (ICU); MAP, mean arterial pressure at admission in ICU; ACT, activated clotting time at admission in ICU; Lac, lactic acid; P/F, PaO_2_/FiO_2_, oxygenation index; NE, neutrophil count; VIS, vasoactive inotrope score; SOFA, Sequential Organ Failure Assessment; 0 h, Patients at admission in ICU after surgery; 24 h, Patients in ICU at 24 h after surgery. ^1^*p* and ^2^*p* < 0.017 indicates statistical significance; ^3^*p* < 0.05 indicates statistical significance. The bolded data indicate statistical significance.

### 3.3 Coagulation Activity in the Patients With and Without ARDS in the ATAAD Cohort

The levels of coagulation factors in the patients with ARDS from the ATAAD group are shown in Tables 3,4. Preoperative D-dimer levels were similar between the non-ARDS and ARDS groups (2403.0 [942.0–7524.5] vs 5302 [1184.0–9419.0] ng/mL, *p* = 0.134). The concentration of the FDPs also tended to rise in the ARDS group before surgery (22.7 [10.2–65.9] vs 49.3 [12.5–80.8] µg/mL, *p* = 0.098). At the end of surgery, D-dimer levels in the ARDS group were significantly higher than those in the non-ARDS group (3326 [2270.0–5989.0] vs 2281.0 [1135.0–3787.5] ng/mL, *p* = 0.017). Twenty-four hours after surgery, there was no significant difference between the groups, although the D-dimer levels were significantly elevated in the ARDS group 48 hours after surgery (2017 [935.0–2955.0] vs. 935.0 [869.5–2617.0] ng/mL, *p* = 0.046). The trend of FDPs levels was consistent with that of the D-dimer levels. In-hospital mortality was significantly higher postoperatively in the patients with ARDS (25.8% vs. 4.2%, *p* = 0.001).

**Table 3.** **Baseline among ARDS and non-ARDS patients within ATAAD cohort**.

Variables		Non-ARDS	ARDS	*p*
		(n = 119)	(n = 31)	
Demographic information and preoperative complications				
	Age (yrs)	48.5 ± 12.0	51.6 ± 9.1	0.179
	Sex (male%)	87 (73.1)	27 (87.1)	0.165
	BMI (kg/m^2^)	27.1 ± 3.8	27.6 ± 3.8	0.526
	Smoking	51 (42.9)	14 (45.2)	0.978
	Alcohol	14 (11.8)	5 (16.1)	0.728
	HTN	101 (84.9)	25 (80.6)	0.766
	Diabetes	10 (8.4)	3 (9.7)	1
	AKI	22 (18.5)	1 (3.2)	0.069
	LD	10 (8.4)	1 (3.2)	0.550
	HF	37 (31.1)	12 (38.7)	0.555
	CLD	3 (2.5)	1 (3.2)	1
	ND	1 (0.8)	0 (0.0)	1
	Prior CS	1 (0.8)	1 (3.2)	0.879
	Shock	5 (4.2)	2 (6.5)	0.959
	AC	12 (10.1)	3 (9.7)	1
	EuroSCORE	5.0 (5.0, 6.5)	5.0 (5.0, 6.0)	0.622
Preoperative laboratory values				
	PaCO_2_	36.7 (33.3, 39.6)	35.6 (33.4, 39.4)	0.492
	PaO_2_	100.0 (80.7, 123.5)	92.6 (69.8, 111.0)	0.138
	Lac	1.4 (1.0, 2.2)	1.9 (1.2, 3.0)	0.094
	PT	12.0 (11.5, 12.6)	11.9 (11.3, 12.7)	0.922
	APTT	30.2 (28.6, 32.2)	31.1 (26.9, 32.4)	0.873
	D-dimer	2403.0 (942.0, 7524.5)	5302.0 (1184.0, 9419.0)	0.134
	FBG	2.3 (1.8, 3.0)	2.0 (1.7, 2.5)	0.100
	FDPs	22.7 (10.2, 65.9)	49.3 (12.5, 80.8)	0.098

*p* < 0.05 indicates statistical significance. The bolded data indicate statistical significance.

**Table 4.** **Intraoperative and postoperative clinical factors among ARDS and non-ARDS patients within ATAAD cohort**.

Variables		Non-ARDS	ARDS	*p*
		(n = 119)	(n = 31)	
Surgery-related variables				
	Surgical duration (h)	8.0 (7.0, 9.0)	8.0 (7.5, 9.3)	0.086
	CPB time (min)	176.0 (158.0, 202.0)	192.0 (162.0, 222.5)	0.078
	DHCA time (min)	19.0 (12.5, 29.0)	24.0 (15.0, 36.5)	0.055
	ACC time (min)	94.0 (84.5, 112.5)	113.0 (93.5, 122.5)	**0.019**
	Heparin input (mL)	10.0 (10.0, 10.0)	10.0 (10.0, 11.0)	0.431
	Plasma input (mL)	0.0 (0.0, 400.0)	0.0 (0.0, 0.0)	0.184
Postoperative laboratory values				
	HR	88.0 (75.0, 98.0)	90.0 (79.5, 103.5)	0.342
	MAP (mm/Hg)	94.0 (83.4, 103.8)	90.5 (78.3, 100.3)	0.256
	ACT	161.0 (147.0, 178.0)	160.0 (149.0, 173.0)	0.705
	PaCO_2_ 0 h	46.5 (42.7, 50.0)	47.3 (42.4, 53.1)	0.462
	PaCO_2_ 24 h	39.4 (35.5, 43.3)	39.4 (34.8, 42.2)	0.279
	PaO_2_ 0 h	110.0 (85.6, 161.5)	109.0 (86.6, 141.0)	0.876
	PaO_2_ 24 h	94.5 (78.6, 117.5)	90.6 (72.3, 110.5)	0.393
	Lac 0 h	2.3 (1.5, 3.3)	2.6 (1.8, 4.0)	0.224
	Lac 24 h	2.2 (1.5, 3.2)	2.2 (1.9, 3.2)	0.202
	P/F 0 h	182.8 (131.1, 273.4)	162.3 (137.3, 235.4)	0.598
	P/F 24 h	213.8 (158.6, 298.8)	188.3 (122.4, 265.0)	0.154
	P/F 48 h	275.7 (193.3, 333.4)	187.3 (129.7, 218.2)	< **0.001**
	NE 24 h	10.2 (7.8, 12.4)	9.5 (7.3, 12.3)	0.590
	NE 48 h	13.9 (11.2, 16.5)	12.3 (10.7, 16.4)	0.415
	PT 0 h	13.2 (12.4, 14.0)	13.3 (12.6, 14.8)	0.288
	PT 24 h	13.0 (12.2, 14.0)	13.1 (12.2, 13.7)	0.688
	PT 48 h	12.3 (11.6, 13.0)	12.5 (11.7, 13.5)	0.328
	APTT 0 h	33.9 (31.0, 40.1)	33.6 (31.9, 40.3)	0.897
	APTT 24 h	30.4 (27.3, 33.7)	30.7 (28.1, 35.7)	0.426
	APTT 48 h	31.0 (27.8, 31.4)	31.0 (27.4, 33.7)	0.825
	D-dimer 0 h	2281.0 (1135.0, 3787.5)	3326.0 (2270.0, 5989.0)	**0.017**
	D-dimer 24 h	2222.0 (1095.0, 3358.5)	2823.0 (1881.5, 3558.5)	0.213
	D-dimer 48 h	935.0 (869.5, 2617.0)	2017.0 (935.0, 2955.0)	**0.046**
	FBG 0 h	2.7 (2.1, 3.6)	2.3 (1.6, 2.9)	**0.013**
	FBG 24 h	3.7 (2.9, 4.5)	3.7 (3.3, 4.5)	0.809
	FBG 48 h	4.2 (3.8, 4.6)	4.2 (3.3, 4.9)	0.696
	FDPs 0 h	16.3 (9.7, 33.3)	33.5 (17.4, 57.9)	**0.008**
	FDPs 24 h	20.8 (11.3, 33.8)	29.6 (17.8, 37.2)	0.128
	FDPs 48 h	8.9 (8.5, 23.6)	15.4 (8.8, 28.1)	**0.037**
	VIS 24 h	6.0 (3.0, 11.0)	10.0 (3.5, 20.0)	0.157
	VIS 48 h	3.0 (0.0, 8.0)	5.0 (3.0, 11.0)	0.054
	SOFA 24 h	10.0 (7.0, 13.0)	11.0 (8.5, 15.0)	0.069
	SOFA 48 h	7.0 (5.0, 11.0)	10.0 (8.0, 14.5)	**0.001**
	MV duration (h)	19.0 (12.0, 61.0)	39.0 (20.0, 108.5)	**0.014**
	ICU duration (h)	65.0 (37.0, 138.0)	106.0 (64.5, 201.0)	**0.027**
	In-hospital mortality (%)	5 (4.2)	8 (25.8)	**0.001**

MV, Mechanical ventilation; 0 h, Patients at admission in ICU after surgery; 24 h, Patients in ICU at 24 h after surgery; 48 h, Patients in ICU at 48 h after surgery. *p* < 0.05 indicates statistical significance. The bolded data indicate statistical significance.

After all coagulation biomarkers were normalized and standardized using log- and z-score transformations, the results indicated that the levels of FDPs at the end of surgery (OR: 2.26, 95% CI: 1.13–4.54; *p* = 0.022) was the independent risk factor for the development of ARDS among the patients with ATAAD (Table 5).

**Table 5.** **Correlation between coagulation activity and postoperative ARDS in patients in three groups**.

Variables	ATAAD				AA				UA			
	Model 1		Model 2		Model 1		Model 2		Model 1		Model 2	
	OR (95% CI)	*p*	OR (95% CI)	*p*	OR (95% CI)	*p*	OR (95% CI)	*p*	OR (95% CI)	*p*	OR (95% CI)	*p*
z-pre-PT	0.93 (0.58, 1.49)	0.756	0.87 (0.45, 1.65)	0.661	1.07 (0.77, 1.49)	0.698	1.42 (0.88, 2.28)	0.151	0.38 (0.10, 1.39)	0.143	0.39 (0.08, 2.00)	0.261
z-pre-APTT	1.02 (0.70, 1.50)	0.900	1.02 (0.66, 1.57)	0.942	0.81 (0.39, 1.70)	0.577	1.41 (0.55, 3.61)	0.474	0.85 (0.43, 1.68)	0.638	0.76 (0.21, 2.72)	0.668
z-pre-D-dimer	1.51 (0.89, 2.59)	0.130	1.39 (0.72, 2.69)	0.328	1.21 (0.67, 2.16)	0.525	1.19 (0.61, 2.33)	0.608	1.63 (0.52, 5.17)	0.403	3.81 (0.56, 25.8)	0.170
z-pre-FBG	0.75 (0.51, 1.09)	0.130	0.76 (0.48, 1.20)	0.237	0.96 (0.60, 1.54)	0.863	0.85 (0.46, 1.56)	0.594	1.67 (0.55, 5.05)	0.364	5.04 (0.69, 36.5)	0.110
z-pre-FDPs	1.54 (0.95, 2.51)	0.083	1.41 (0.78, 2.55)	0.252	1.16 (0.60, 2.23)	0.659	0.97 (0.44, 2.16)	0.943	1.67 (0.41, 6.75)	0.472	18.8 (1.08, 32.8)	0.044
z-PT 0 h	1.05 (0.75, 1.47)	0.782	1.09 (0.76, 1.57)	0.642	1.84 (0.82, 4.14)	0.139	2.38 (0.89, 6.36)	0.085	0.84 (0.34, 2.06)	0.705	1.31 (0.36, 4.85)	0.684
z-PT 24 h	1.08 (0.72, 1.47)	0.719	1.42 (0.87, 2.31)	0.161	1.13 (0.68, 1.87)	0.636	1.33 (0.70, 2.50)	0.384	0.87 (0.47, 1.62)	0.670	0.82 (0.36, 1.86)	0.629
z-PT 48 h	1.25 (0.86, 1.81)	0.236	1.35 (0.87, 2.09)	0.175	/		/		/		/	
z-APTT 0 h	1.02 (0.63, 1.65)	0.951	1.07 (0.61, 1.87)	0.820	1.15 (0.71, 1.87)	0.558	1.04 (0.58, 1.88)	0.893	1.28 (0.59, 2.78)	0.540	0.97 (0.37, 2.55)	0.958
z-APTT 24 h	1.36 (0.84, 2.21)	0.210	1.41 (0.78, 2.54)	0.257	0.77 (0.33, 1.81)	0.551	0.49 (0.19, 1.23)	0.129	1.16 (0.73, 1.82)	0.530	0.99 (0.45, 2.17)	0.980
z-APTT 48 h	1.00 (0.67, 1.48)	0.991	1.02 (0.66, 1.57)	0.933	/		/		/		/	
z-D-dimer 0 h	1.95 (1.06, 3.59)	**0.032**	2.03 (0.96, 4.28)	0.063	2.04 (1.11, 3.50)	**0.008**	1.63 (0.89, 2.98)	0.111	1.62 (0.59, 4.43)	0.346	2.82 (0.53, 15.2)	0.230
z-D-dimer 24 h	1.26 (0.68, 2.33)	0.455	1.24 (0.63, 2.47)	0.533	1.73 (0.99, 3.01)	0.053	1.41 (0.72, 2.74)	0.319	1.59 (0.63, 4.02)	0.362	1.95 (0.65, 5.88)	0.234
z-D-dimer 48 h	1.57 (1.06, 2.32)	**0.026**	1.55 (0.97, 2.47)	0.066	/		/		/		/	
z-FBG 0 h	0.43 (0.23, 0.81)	**0.008**	0.40 (0.19, 0.83)	**0.014**	0.58 (0.26, 1.32)	0.196	0.46 (0.17, 1.22)	0.117	1.05 (0.74, 1.50)	0.768	1.10 (0.50, 2.44)	0.814
z-FBG 24 h	1.02 (0.63, 1.65)	0.934	0.99 (0.59, 1.68)	0.978	0.64 (0.33, 1.23)	0.181	0.60 (0.28, 1.29)	0.189	1.18 (0.67, 2.09)	0.559	0.99 (0.46, 2.11)	0.979
z-FBG 48 h	0.88 (0.60, 1.30)	0.521	0.85 (0.51, 1.39)	0.510	/		/		/		/	
z-FDPs 0 h	2.05 (1.17, 3.60)	**0.012**	2.26 (1.13, 4.54)	**0.022**	1.92 (1.19, 3.09)	**0.008**	1.60 (0.91, 2.81)	0.105	1.12 (0.27, 4.65)	0.875	1.83 (0.22, 15.3)	0.575
z-FDPs 24 h	1.45 (0.78, 2.69)	0.235	1.42 (0.70, 2.84)	0.329	1.56 (0.96, 2.55)	0.075	1.40 (0.74, 2.65)	0.302	1.79 (0.61, 5.25)	0.287	2.37 (0.52, 10.8)	0.265
z-FDPs 48 h	1.50 (1.02, 2.21)	**0.040**	1.51 (0.95, 2.40)	0.080	/		/		/		/	

z: All biomarkers were normalized and standardized by log transformation and z-score transformation. Model 1: Univariate logistic regression. Model 2: Multivariate logistic regression. Adjusted covariates: age, sex, BMI, Hypertension, Diabetes, AKI, LD, CLD, ND, HF, Smoking, Alcohol, Prior CS, preoperative AST, preoperative oxygenation index. These covariates that showed significant differences between the ARDS group and Non-ARDS group in the baseline characteristics (**Supplementary Table 1**). Pre, preoperative. *p* < 0.05 indicates statistical significance. The bolded data indicate statistical significance.

### 3.4 Serum Concentrations of Coagulation Biomarkers

**Fig. 1.**
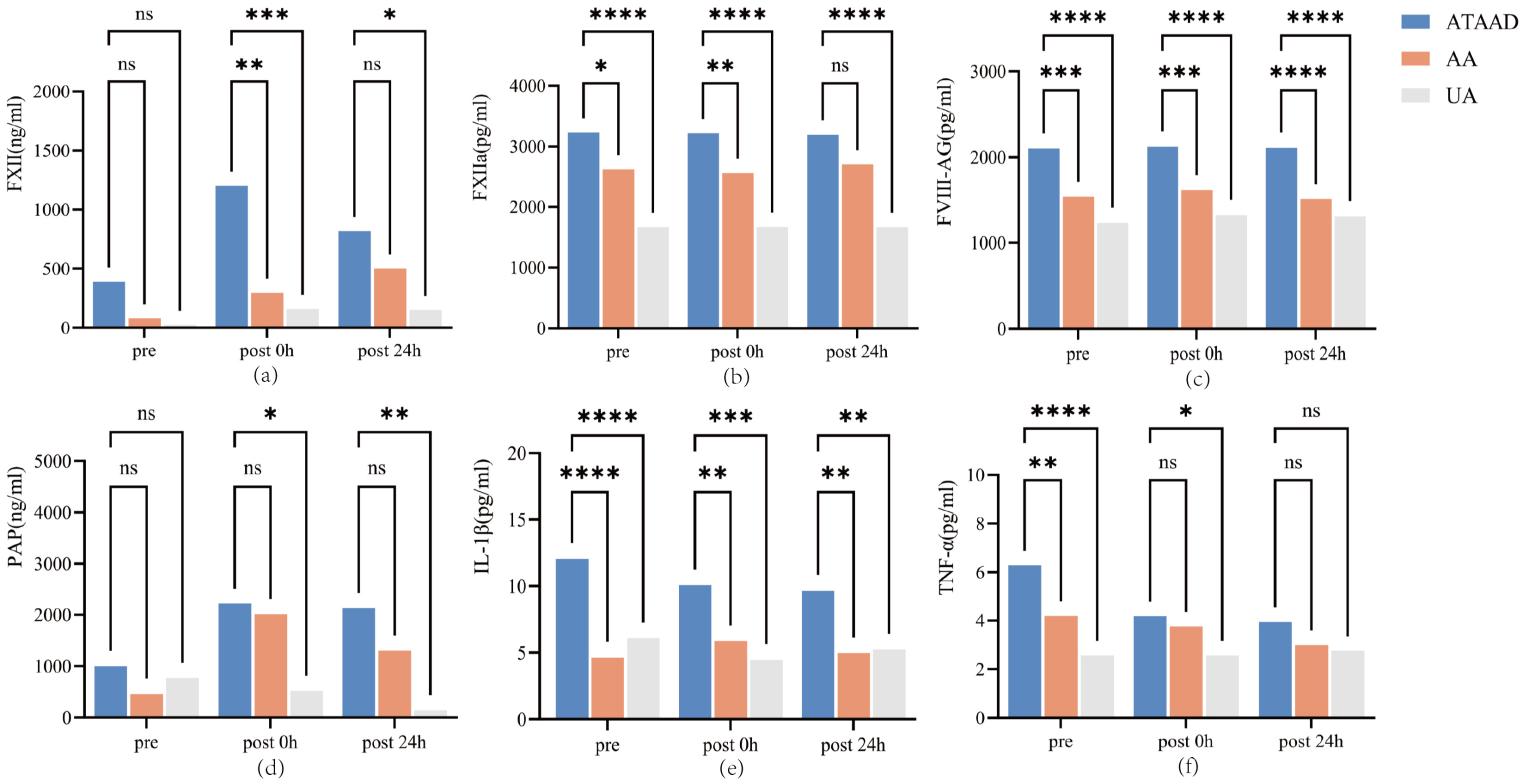
**The serum concentration of coagulation and inflammation biomarkers among three groups**. (a) FXII levels: Intergroup comparisons across time points; (b) FXIIa levels: Intergroup comparisons across time points; (c) FVIII-Ag levels: Intergroup comparisons across time points; (d) PAP levels: Intergroup comparisons across time points; (e) IL-1β levels: Intergroup comparisons across time points; (f) TNFα levels: Intergroup comparisons across time points. FXII, factor XII; FXIIa, factor XIIa; FVIII-Ag, factor VIII-related antigen; PAP, plasmin-antiplasmin complex; IL-1β, interleukin one beta, and TNFα: tumor necrosis factor-alpha. Pre, immediately prior to anesthesia induction; post 0 h, immediately after surgery; post 24 h, 24 h after surgery. ****: *p* < 0.0001; ***: *p* < 0.001; **: *p* < 0.005; *: *p* < 0.05; ns: *p* > 0.05.

The effects of the different groups and phases on the concentrations of FXII, FXIIa, FVIII-Ag, and PAP are presented in Fig. 1. Preoperatively (immediately prior to anesthesia induction), compared with those in the other two groups, patients with ATAAD presented with significantly higher levels of FXIIa (*p* = 0.014 and *p* < 0.0001 for ATAAD vs. AA and for ATAAD vs. UA groups, respectively) and FVIII-Ag (*p* = 0.0002 and *p* < 0.0001 for ATAAD vs. AA and for ATAAD vs. UA groups, respectively). After surgery, FXII, FXIIa, and FVIII-Ag levels were significantly higher in the ATAAD group compared to those in the other groups (Fig. 1a–c), whereas the perioperative PAP levels were similar between the ATAAD and AA groups (*p* > 0.05, Fig. 1d).

**Fig. 2.**
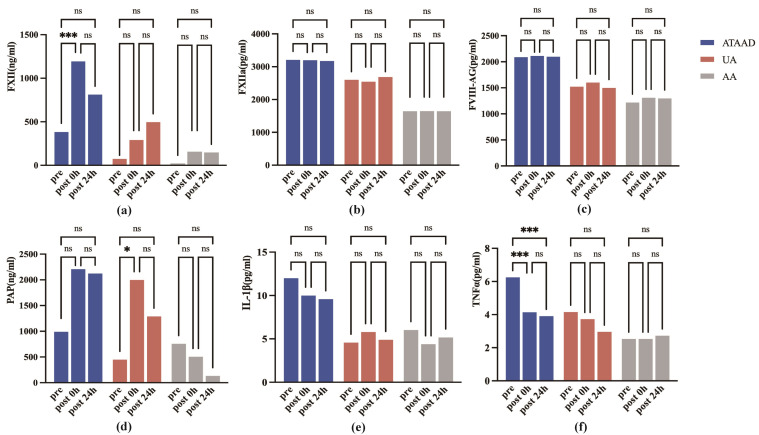
**The serum concentration of coagulation and inflammation biomarkers at different time points for the three groups**. (a) FXII levels: Intragroup temporal variations; (b) FXIIa levels: Intragroup temporal variations; (c) FVIII-Ag levels: Intragroup temporal variations; (d) PAP levels: Intragroup temporal variations; (e) IL-1β levels: Intragroup temporal variations; (f) TNFα levels: Intragroup temporal variations. FXII, factor XII; FXIIa, factor XIIa; FVIII-Ag, factor VIII-related antigen; PAP, plasmin-antiplasmin complex; IL-1β, interleukin one beta, and TNFα, tumor necrosis factor-alpha. Pre, immediately prior to anesthesia induction; post 0 h, immediately after surgery; post 24 h, 24 h after surgery. ***: *p* < 0.001; *: *p* < 0.05; ns: *p* > 0.05.

After conducting statistical analysis on the same indicators of the same group at different time points, it was found that the FXII level at 0 h after was significantly higher than that immediately prior to anesthesia induction (*p* < 0.001, Fig. 2a) and the TNF level immediately prior to anesthesia induction was significantly higher than that after surgery (*p* < 0.001, Fig. 2f) in patients with ATAAD. The level of PAP at immediately prior to anesthesia induction was significantly higher than that after surgery (*p* < 0.05, Fig. 2d) in patients with AA. The levels of other biomarkers did not show significant differences at each time point (Fig. 2).

**Fig. 3.**
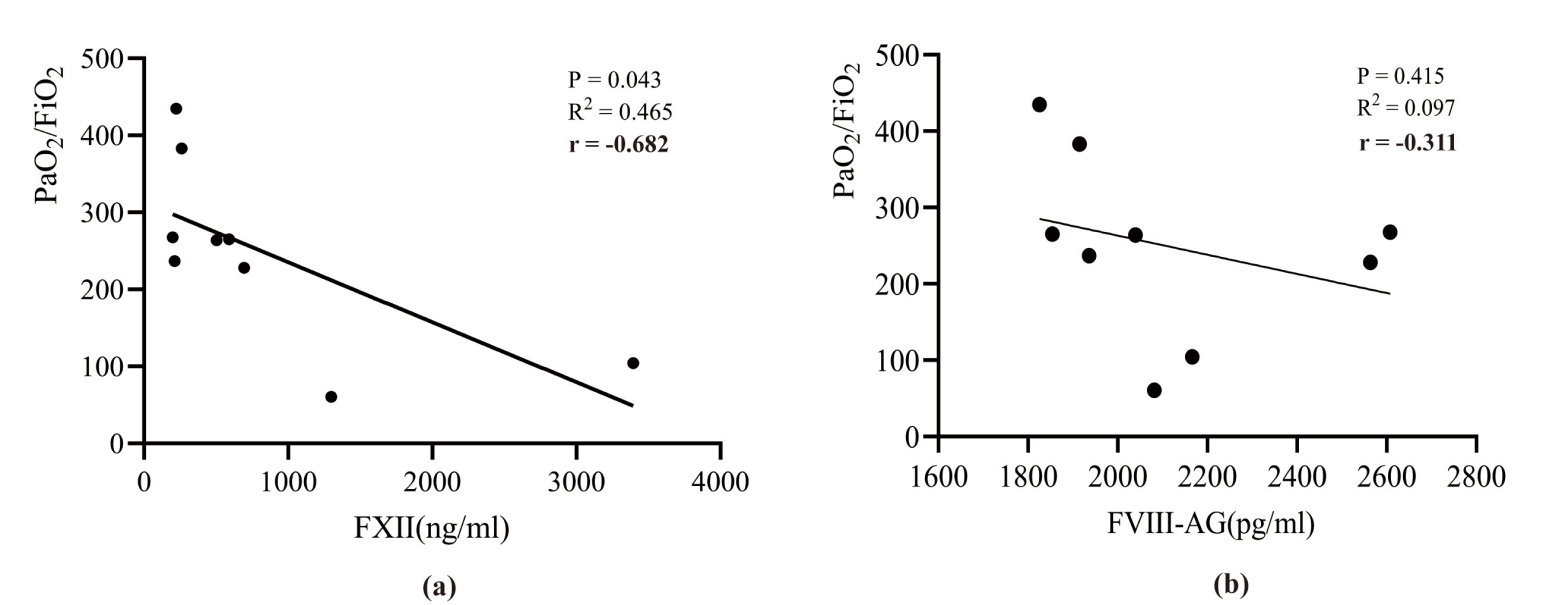
**The correlation between coagulation factors and oxygenation index in patients with ATAAD**. (a) Association of FXII concentration with PaO_2_/FiO_2_; (b) Association of FVIII-Ag concentration with PaO_2_/FiO_2_. PaO_2_/FiO_2_, oxygenation index; FXII, factor XII; FVIII-Ag, factor VIII-related antigen.

There was a negative correlation between FXII levels and the oxygenation index 24 h post-surgery (Pearson correlation coefficient r = –0.682, *p* = 0.043), whereas no significant correlations were observed for the other markers or at other time points (Fig. 3). However, a trend was observed in which the oxygenation index decreased as FVIII-Ag levels increased, suggesting that FVIII-Ag may affect lung function.

## 4. Discussion

The aim of this study was to investigate if coagulation activity could be induced by ATAAD onset or CPB, and to determine the impact of coagulation activity on the development of post-operative ARDS. The results revealed significantly elevated serum concentrations of perioperative coagulation biomarkers in patients with ATAAD who had undergone surgery involving CPB. Meanwhile, the results indicate that the elevation of coagulation biomarkers is a risk factor for the occurrence of ARDS.

### 4.1 Activation of Coagulation System in Patients With ATAAD

The present study, which used a control group of patients with AA, verified that ATAAD itself can significantly increase the activation of the coagulation system. ATAAD is caused by injury to the aortic intima, where blood gains access to the false lumen formed between the intima and media. The activation of the coagulation system could be induced by contact between blood and the false lumen and the release of coagulant material from the aortic wall into the systemic circulation [26, 27].

Previous studies [28, 29] have demonstrated that ATAAD and the surgical interventions to treat it can cause an increase in the levels of biomarkers that induce the activation of coagulation. The preoperative levels of FDPs and D-dimer become sharply elevated in patients with ATAAD [27, 30]. An increased D-dimer level reflects both the augmented formation of fibrin as well as the degree of subsequent fibrinolysis, and it has been shown to be associated with postoperative adverse events in patients with ATAAD [31, 32]. However, a small number of studies [33, 34] have compared the perioperative coagulation function of patients with ATAAD undergoing emergency surgery and that of patients with AA undergoing elective aortic surgery.

In the present study, intergroup comparisons were performed between patients with ATAAD and those with AA or UA; the results showed that the preoperative (immediately prior to anesthesia induction) levels of FXIIa and FVIII-Ag were significantly higher in the ATAAD group than in the other two groups, whereas the preoperative fibrinogen level was significantly lower, indicating that patients with ATAAD consumed more fibrinogen before surgery. Although patients experiencing chronic aneurysms also presented with higher D-dimer levels, the upregulation was more pronounced in patients with ATAAD. However, whether there is a causal relationship between ATAAD itself and ARDS still requires further research to verify.

### 4.2 Activation of Coagulation System in Patients After CPB Surgery

In this study, the levels of FXII, FXIIa, FVIII-Ag, D-dimer, and FDPs were elevated in patients with ATAAD and AA, and the elevation was more pronounced in the former group. Meanwhile, the CPB duration was significantly prolonged in ATAAD group. We hypothesize that the coagulation disorder after CPB is related to the activation of the “contact system”.

The “contact system” is a plasma protease cascade that plays an important role in the activation of the coagulation systems; it includes FXII, FXIIa, factor XI (FXI), high-molecular-weight kininogen (HMWK), and prekallikrein, which are associated proteins in the plasma [17]. Contact system activity increases dramatically as blood comes into contact with the artificial materials comprising CPB circuits [35]. Mechanistically, FXII cleaves itself upon contact with various anionic surfaces, resulting in the production of FXIIa, which subsequently converts prekallikrein into active kallikrein. In the plasma, kallikrein generates a positive feedback loop by cleaving additional FXII, which produces more bradykinin from HMWK [17]. Previous studies have shown that active thrombin can induce the release of tissue plasminogen activator in vitro, with bradykinin potentially serving as the predominant stimulus [36, 37]. Meanwhile, the binding of soluble fibrin to CPB circuits promotes plasminogen activation [38, 39].

Previous studies have reported that coagulation disorders can be deteriorated by use of CPB during surgery, especially in conditions that promote deep hypothermia [20, 33, 40, 41]. For example, some groups have reported that CPB causes an increase in the levels of FXIIa and FDPs [17, 20]. A study conducted by Boisclair *et al*. [42] suggested that FXIIa can serve as a marker of contact system activation in patients undergoing CPB procedures. In addition to producing kallikrein, FXIIa has been reported to convert FXI into FXIa, thereby initiating the activation of the intrinsic pathway [20]. Therefore, the contact system exacerbates the development of coagulation system disorders when CPB is performed, and this effect may be related to the length of time required to complete CPB procedures. FXIIa, D-dimer and FDPs may serve as early biomarkers for identifying high-risk ARDS patients. We should also pay attention to the coagulation system of patients with prolonged CPB duration.

### 4.3 Correlation Between the Activation of Coagulation and Postoperative ARDS

In the ATAAD group, the concentrations of D-dimer and FDPs increased immediately after surgery in the patients who developed ARDS, indicating that coagulation activity was elevated; however, the PAP concentration did not differ between the ATAAD and AA groups and was lower in patients with hypoxemia. The pathophysiology of ARDS is complex, and the underlying mechanisms have yet to be fully elucidated, especially during acute critical illness [43].

Matthay *et al*. [44] have speculated that one of the mechanisms underlying ARDS pathogenesis involves the activation and dysregulation of coagulation, both in the lungs and systemically. As a result, increased fibrinolytic activity may be insufficient to counteract the amplified coagulation activity in patients with ARDS. This disequilibrium may lead to fibrin deposition along the denuded alveolar basement membrane, prompting hyaline membrane formation; such lesions can, in turn, decrease lung compliance and increase inspiratory pressures [43]. In addition, the activation of procoagulant pathways may cause microvascular thromboses in the lungs; the increased amount of dead space reduces blood flow and subsequently influences gas exchange in those with ARDS [43, 45, 46]. In this study, the oxygenation index was negatively correlated with the concentration of FXII and was positively correlated with the concentration of PAP 24 hours post-surgery, manifesting as a change in coagulation activity that may be associated with ARDS. However, given the limited sample size, additional research is warranted to validate these findings.

The concentrations of the pro-inflammatory cytokines IL-1β and TNFα were also significantly elevated in the ATAAD group. Correlations between ALI and inflammatory responses have been extensively investigated [22, 47]. Both local and systemic acute inflammation are prominent features of ARDS [43], and previous studies have reported that contact with the components of bypass circuits and ischemia–reperfusion injury may upregulate the expression of inflammatory cytokines, thereby leading to the release of coagulation factors [48, 49]. Meanwhile, both FXII and contact activation may induce bradykinin release and complement system activation, further promoting the systemic inflammatory response [48, 49, 50]. These findings indicated that specific interactions between coagulation and inflammation pathways contribute to lung injury.

To date, there has been little evidence to support the possibility that coagulation activation at an early stage following cardiac surgery can increase the likelihood of ARDS development, especially in patients with ATAAD. One of the strengths of this study was the detailed comparison that was performed of the relationships between coagulation disorders and postoperative ARDS between the three groups; however, there were some limitations. Firstly, the study population was derived from a single center, and it was not possible to exclude other factors that may have affected oxygenation; further validation is required with a larger cohort. Secondly, ELISAs were used to test serum samples from just nine randomly selected patients in each group. Future investigations should examine other markers of coagulation and fibrinolysis in larger cohorts. Thirdly, local coagulation and inflammatory activity are also important, as they can influence the occurrence of hypoxemia; further analysis of the coagulation biomarkers will be performed based on bronchoalveolar lavage fluid.

## 5. Conclusion

In conclusion, the ATAAD- or CPB-induced activation of coagulation pathways can affect gas exchange in the lungs. The early increase in the levels of FXII, FXIIa, D-dimer, and FDPs and the decrease in PAP may play an important role in the mechanisms through which patients with ATAAD develop ARDS following surgical procedures involving CPB. This study offers new insights into the clinical treatment of ARDS using aggressive anticoagulant therapies. Further studies are needed to elucidate the underlying cellular and molecular mechanisms through which the activation of coagulation cascades drives the development of postoperative ARDS in patients with ATAAD.

## Data Availability

The datasets used and/or analyzed during the current study are available from the corresponding author on reasonable request.

## Abbreviations

AA, aortic aneurysm; ACI, acute lung injury; APTT, activated partial thromboplastin time; ARDS, acute respiratory distress syndrome; AST, aspartate aminotransferase; ATAAD, acute type A aortic dissection; BIC, Bayesian information criterion; BMI, body mass index; CIs, confidence intervals; CKD, chronic kidney disease; CLD, chronic liver disease; CPB, cardiopulmonary bypass; CS, cardiac surgery; CT, computed tomography; DHCA, deep hypothermic circulatory arrest; ELISA, enzyme-linked immunosorbent assay; ESICM, European Society of Intensive Care Medicine; EuroSCORE, European System for Cardiac Operative Risk Evaluation; FXI, factor XI; FXII, factor XII; FXIIa, factor XIIa; FVIII-Ag, factor VIII-related antigen; HF, heart failure; HMWK, high-molecular-weight kininogen; HTN, hypertension; ICU, intensive care unit; IL-1β, interleukin one beta; ND, neurological dysfunction; OPCABG, off-pump coronary artery bypass grafting; ORs, odds ratios; PAP, plasmin-α2-antiplasmin complex; TNF-α, tumor necrosis factor alpha; UA, unstable angina; VIS, vasoactive inotrope score.

## Supplementary Material

Supplementary material associated with this article can be found, in the online version, at https://doi.org/10.31083/RCM36372.
