# Supplementary material for: Coagulation Disorders in Patients With Acute Respiratory Distress Syndrome Following Acute Aortic Dissection: A Prospective Observational Study
Source: Rev Cardiovasc Med. 2025 Aug 25;26(8):36372. doi: 10.31083/RCM36372 (PMC12415743; doi:10.31083/RCM36372)
Supplement: Supplementary file 1 [file 2153-8174-26-8-36372-s1.docx]

**Tables**

**Supplemental Table 1** **Baseline among ARDS and non-ARDS patients.**

| Variables | Non-ARDS | ARDS | P |
| --- | --- | --- | --- |
|  | **(n=388)** | **(n=62)** |  |
| Demographic information and preoperative complications | | | |
| Age(yrs) | 55.5±12.8 | 56.9±10.7 | 0.412 |
| Sex(male%) | 263 (67.8) | 43 (69.4) | 0.921 |
| BMI (kg/m^2^) | 26.0±3.6 | 26.7±3.4 | 0.180 |
| Smoking | 157 (40.5) | 29 (46.8) | 0.425 |
| Alcohol | 80 (20.6) | 14 (22.6) | 0.853 |
| HTN | 263 (67.8) | 47 (75.8) | 0.263 |
| Diabetes | 72 (18.6) | 7 (11.3) | 0.224 |
| AKI | 31 (8.0) | 4 (6.5) | 0.869 |
| LD | 18 (4.6) | 4 (6.5) | 0.766 |
| HF | 170 (43.8) | 24 (38.7) | 0.538 |
| CLD | 9 (2.3) | 1 (1.6) | 1 |
| ND | 6 (1.5) | 3 (4.8) | 0.218 |
| Prior CS | 13 (3.4) | 3 (4.8) | 0.827 |
| Shock | 5(1.3) | 2 (3.2) | 0.554 |
| AC | 13 (3.4) | 3 (4.8) | 0.827 |
| EuroSCORE | 4.0(3.0, 6.0) | 5.0(5.0, 6.0) | **<0.001** |
| Preoperative laboratory values | | | |
| AST(U/L) | 19.0(15.0, 25.0) | 22.0(17.3, 26.0) | **0.037** |
| PaCO_2_ (mmHg) | 34.9(32.5, 37.8) | 34.9(31.6, 38.1) | 0.604 |
| PaO_2_ (mmHg) | 90.9(81.2, 107.0) | 89.0(74.7, 109.0) | 0.191 |
| Lac (mmol/l) | 1.6(1.2, 2.0) | 1.6(1.1, 2.5) | 0.158 |
| P/F | 379.6(327.4, 432.4) | 356.3(288.5, 390.6) | **0.001** |
| PT | 11.5(11.0, 12.1) | 11.5(11.1, 12.5) | 0.502 |
| APTT | 31.0(29.1, 33.1) | 30.9(27.9, 32.7) | 0.420 |
| D-dimer(ng/ml) | 197.5(81.0, 1537.3) | 897.5(212.8, 5388.3) | **0.001** |
| FBG(g/L) | 2.8(2.4, 3.4) | 2.6(2.0, 3.2) | **0.040** |
| FDPs(μg/ml) | 1.5(0.7, 13.1) | 8.2(1.2, 49.4) | **0.001** |

HTN: Hypertension; AKI: Acute kidney disease; LD: Liver dysfunction; HF: Heart failure; CLD: Chronic lung disease; CS: Cardiac surgery; ND: Neurological dysfunction; AC: Altered consciousness; EuroSCORE: European System for Cardiac Operative Risk Evaluation; FBG: fibrinogen; FDPs: fibrinogen degradation products.

**Supplemental Table 2 Intraoperative and postoperative clinical factors among ARDS and non-ARDS patients.**

| Variables | Non-ARDS | ARDS | P |
| --- | --- | --- | --- |
|  | **(n=388)** | **(n=62)** |  |
| Surgery-related variables | | | |
| Surgical duration(h) | 6.5(5.0,8.0) | 8.0(6.0, 9.0) | **0.001** |
| CPB time(min) | 133.0(0.0, 172.3) | 170.0(119.5, 203.0) | **<0.001** |
| DHCA time(min) | 0.0(0.0, 16.0) | 4.0(0.0, 23.5) | **0.012** |
| ACC time(min) | 73.0(0.0, 95.0) | 95.5(65.3, 116.8) | **<0.001** |
| Heparin input(ml) | 8.0(0.0, 10.0) | 10.0(6.3, 10.0) | **0.001** |
| Plasma input(ml) | 0.0(0.0, 0.0) | 0.0(0.0, 0.0) | 0.673 |
| Postoperative laboratory values | | | |
| HR | 82.0(74.0, 90.0) | 86.5(78.3, 97.5) | **0.014** |
| MAP (mm/Hg) | 84.5(76.0, 92.5) | 86.4(76.1, 96.1) | 0.527 |
| ACT | 154.0(143.0, 166.0) | 156.5(147.0, 165.0) | 0.391 |
| PaCO_2_(mm/Hg) 0h | 42.9(38.3, 46.8) | 44.1(40.4, 48.7) | 0.064 |
| PaCO_2_(mm/Hg) 24h | 39.4(36.1, 42.2) | 39.5(35.0, 42.3) | 0.921 |
| PaO_2_(mm/Hg) 0h | 145.0(99.9, 198.3) | 117.5(90.3, 142.3) | **0.001** |
| PaO_2_(mm/Hg) 24h | 112.0(90.0, 147.9) | 105.0(81.4, 130.8) | **0.036** |
| Lac(mmol/l) 0h | 1.6(1.1, 2.5) | 2.2(1.4, 3.6) | **0.001** |
| Lac(mmol/l) 24h | 2.2(1.5, 3.4) | 2.3(1.7, 3.6) | 0.242 |
| P/F 0h (%) | 253.7(166.7, 351.7) | 195.9(145.6, 237.8) | **<0.001** |
| P/F 24h (%) | 277.5(208.2, 357.5) | 228.4(169.9, 277.2) | **<0.001** |
| NE (x10^9^/L) 24h | 9.82(7.8, 12.6) | 9.2(7.1, 11.8) | 0.175 |
| NE (x10^9^/L) 48h | 13.1(10.1, 16.6) | 11.9(9.3, 14.9) | 0.101 |
| PT 0h | 13.2(12.4, 14.1) | 13.5(12.6, 14.6) | 0.195 |
| PT 24h | 13.1(12.4, 14.2) | 13.2(12.5, 13.9) | 0.756 |
| APTT 0h | 32.2(29.7, 37.4) | 33.3(31.5, 38.1) | 0.157 |
| APTT 24h | 30.8(28.2, 34.0) | 31.2(28.6, 34.7) | 0.494 |
| D-dimer(ng/ml) 0h | 627.0(210.8, 2306.8) | 2661.0(625.8, 5309.5) | **<0.001** |
| D-dimer(ng/ml) 24h | 891.0(265.0, 2166.5) | 2027.5(729.5, 3245.8) | **<0.001** |
| FBG(g/L) 0h | 2.4(1.9, 3.1) | 2.4(1.6, 2.9) | 0.129 |
| FBG(g/L) 24h | 3.1(2.5, 4.0) | 3.4(2.4, 4.2) | 0.538 |
| FDPs(μg/ml) 0h | 4.8(1.5, 16.3) | 21.0(6.0, 46.9) | **<0.001** |
| FDPs(μg/ml) 24h | 7.7(2.3, 18.5) | 18.0(6.7, 34.7) | **<0.001** |
| VIS 24h | 6.0(3.0, 11.3) | 8.0(4.0, 15.8) | 0.066 |
| VIS 48h | 3.0(0.0, 8.0) | 4.5(0.0, 10.0) | 0.111 |
| SOFA 24h | 7.0(5.0, 9.0) | 9.0(6.0, 12.0) | **0.001** |
| SOFA48h | 5.0(3.0, 7.0) | 8.0(5.0, 10.0) | **<0.001** |

CPB: Cardiopulmonary bypass; DHCA: Deep Hypothermic Circulatory Arrest; ACC: Aortic cross-clamp; HR: Heart rate at admission in intensive care unit (ICU); MAP: Mean arterial pressure at admission in ICU; ACT: Activated clotting time at admission in ICU; Lac: Lactic acid; P/F: PaO_2_/FiO_2,_ oxygenation index; NE: neutrophil count; FBG: fibrinogen; FDPs: fibrinogen degradation products; VIS: vasoactive inotrope score ; SOFA: Sequential Organ Failure Assessment ; MV: Mechanical ventilation; 0h: Patients at admission in ICU after surgery; 24h: Patients in ICU at 24h after surgery; 48h: Patients in ICU at 48h after surgery.

**Supplemental Table 3** **Multivariate logistic regression analysis between ARDS and non-ARDS patients.**

| **Variables** | **Model 1** | | **Model 2** | | **Collinearity test** |
| --- | --- | --- | --- | --- | --- |
|  | **OR (95% CI)** | **P** | **OR (95% CI)** | **P** | **VIF** |
| **z-pre-PT** | 1.07(0.83,1.38) | 0.590 | 1.09 (0.80, 1.48) | 0.604 | 1.457 |
| **z-pre-APTT** | 0.90(0.67,1.21) | 0.481 | 0.89 (0.64, 1.24) | 0.495 | 1.071 |
| **z-pre-D-dimer** | 1.60(1.23,2.08) | **0.001** | 1.78 (1.31, 2.42) | **<0.001** | 1.382 |
| **z-pre-FBG** | 0.73(0.54,0.97) | **0.032** | 0.69 (0.50, 0.96) | **0.025** | 1.163 |
| **z-pre-FDPs** | 1.58(1.23,2.02) | **0.001** | 1.76 (1.31, 2.36) | **<0.001** | 1.418 |
| **z-PT 0h** | 1.18(0.95,1.45) | 0.128 | 1.21 (0.97, 1.52) | 0.094 | 1.040 |
| **z-PT 24h** | 0.95(0.72,1.24) | 0.686 | 0.95 (0.72, 1.27) | 0.738 | 1.049 |
| **z-APTT 0h** | 1.19(0.89,1.60) | 0.234 | 1.18 (0.86, 1.62) | 0.298 | 1.050 |
| **z-APTT 24h** | 1.13(0.86,1.51) | 0.380 | 1.08 (0.80, 1.44) | 0.616 | 1.050 |
| **z-D-dimer 0h** | 1.99(1.49,2.66) | **<0.001** | 2.17 (1.58, 2.99) | **<0.001** | 1.166 |
| **z-D-dimer 24h** | 1.73(1.29,2.30) | **<0.001** | 1.89 (1.37, 2.60) | **<0.001** | 1.228 |
| **z-FBG 0h** | 0.71(0.48,1.05) | **0.082** | 0.65 (0.43, 0.99) | **0.014** | 1.080 |
| **z-FBG 24h** | 1.03(0.79,1.35) | 0.818 | 1.00 (0.74, 1.35) | 0.989 | 1.101 |
| **z-FDPs 0h** | 1.93(1.48,2.53) | **<0.001** | 2.11 (1.57, 2.84) | **<0.001** | 1.171 |
| **z-FDPs 24h** | 1.72(1.31,2.26) | **<0.001** | 1.85 (1.37, 2.52) | **<0.001** | 1.207 |

FBG: fibrinogen; FDPs: fibrinogen degradation products.

Model 1: Univariate logistic regression.

Model 2: Multivariate logistic regression. Adjusted covariates: age, sex, BMI, Hypertension, Diabetes, AKI, LD, CLD, ND, HF, Smoking, Alcohol, Prior CS, preoperative AST, preoperative oxygenation index. These covariates that showed significant differences between the ARDS group and Non-ARDS group in the baseline characteristics (Supplemental Table S1). Pre: preoperative
